# GeneSpeed Beta Cell: An Online Genomics Data Repository and Analysis Resource Tailored for the Islet Cell Biologist

**DOI:** 10.1155/2008/312060

**Published:** 2008-09-09

**Authors:** Nayeem Quayum, Alecksandr Kutchma, Suparna A. Sarkar, Kirstine Juhl, Gerard Gradwohl, Georg Mellitzer, John C. Hutton, Jan Jensen

**Affiliations:** ^1^Barbara Davis Center for Childhood Diabetes, University of Colorado, HSC., 1775 Ursula Street, B140, Aurora, CO 80045, USA; ^2^Development and Physiopathology of the Intestine and Pancreas, Inserm-ULP Unit 682, 3 avenue Molière, Strasbourg 67200, France; ^3^Lerner Research Institute, Department of Stem Cell and Regenerative medicine Cleveland Clinic, 9500 Euclid avenue, Cleveland, OH 44195, USA

## Abstract

*Objective*. We here describe the development of a freely available online database resource, GeneSpeed Beta Cell, which has been created for the pancreatic islet and pancreatic developmental biology investigator community. *Research Design and Methods*. We have developed GeneSpeed Beta Cell as a separate component of the GeneSpeed database, providing a genomics-type data repository of pancreas and islet-relevant datasets interlinked with the domain-oriented GeneSpeed database. *Results*. GeneSpeed Beta Cell allows the query of multiple published and unpublished select genomics datasets in a simultaneous fashion (multiexperiment viewing) and is capable of defining intersection results from precomputed analysis of such datasets (multidimensional querying). Combined with the protein-domain categorization/assembly toolbox provided by the GeneSpeed database, the user is able to define spatial expression constraints of select gene lists in a relatively rigid fashion within the pancreatic expression space. We provide several demonstration case studies of relevance to islet cell biology and development of the pancreas that provide novel insight into islet biology. *Conclusions*. The combination of an exhaustive domain-based compilation of the transcriptome with gene array data of interest to the islet biologist affords novel methods for multidimensional querying between individual datasets in a rapid fashion, presently not available elsewhere.

## 1. INTRODUCTION

Genomics is playing a growing role in almost any biological experimentation. Based on presently available commercial expression array technologies, an investigator is given almost full-genome coverage of transcriptional changes that provides for novel methods for gene identification and validation. However, exhaustive data mining from genomics datasets is cumbersome, and to a large extent is outside the expertise of the individual experimenter. The greatest strength in genomics data analysis stems from multidimensional analysis, as such orthogonal comparison can bring out biologically relevant information not extractable from the individual datasets alone. However, such multidimensional querying is often advised against, as individual genomics experiments are performed in different laboratories, using dissimilar methodologies. Such array data should not be uploaded and analyzed concomitantly in the available software data analysis programs commonly used. Prudent analysis of multiexperimental results would therefore call for individual data analysis of experimental sets, and only parse for intersections/exclusions within the resulting gene lists. This is possible through genomics analysis platforms using separate gene list saving. However, the process is burdened by the fact that the analysis of a relevant dataset for orthogonal querying requires the identification of the existence of the data, upload, normalization, and scaling of individual DNA chip scan files and thereafter selecting and executing a valid analysis for the particular dataset, followed by results storage. In practice, this is time consuming, and too overwhelming, for most biologists.

**Figure 1:**
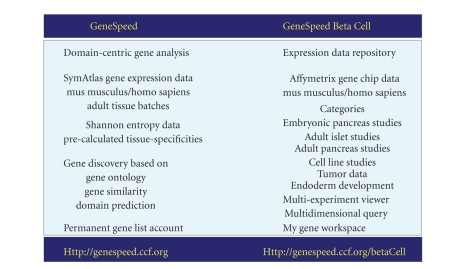
GeneSpeed and GeneSpeed Beta Cell comparison.

In the islet and islet developmental biology research fields, a continuously growing set of public genomics data is becoming available. Also, initial problems in both genomics chip design and experimental execution are gradually being overcome. It is therefore appreciated that a large, generally untapped resource is provided by genomics analyses performed in islet-focused laboratories around the world. Two online databases, T1dbase [1] and EpconDb [2], contain genome data repository components within their sites. However, they do not provide advanced multiexperimental querying options that would allow generation of gene lists between experiments. Acknowledging this, we set forth to create a resource that would consolidate diabetes-research relevant genomics data and allow rapid multidimensional analysis between such datasets. To do this, we created an online genomics data repository, which we term GeneSpeed Beta Cell. This was developed as an additional component of the GeneSpeed resource [3], see Figure 1. GeneSpeed Beta Cell (http://genespeed.ccf.org/betaCell/) contains two forms of data: normalized and similarly scaled genomics data relevant for the islet or pancreatic developmental biologist. Secondly, it contains precalculated analyses, which include pairwise and self-organizing neural network clustering results applied to relevant data series. On the analysis side, GeneSpeed Beta Cell provides “My Gene Workspace” where gene list overlap can be evaluated. It also provides access to any search parameter in the GeneSpeed environment, including precalculated data on tissue specificity (Shannon entropy) and wide-tissue batch expression queries. Together, the unified environment within the GeneSpeed database provides for some unique capacities not found elsewhere. We here describe the use of GeneSpeed Beta Cell by addressing a set of novel, and biologically relevant, questions appealing to the islet biologist.

## 2. GENESPEED BETA CELL DATABASE STRUCTURE AND NAVIGATION

### 2.1. Multiexperimental viewing

**Figure 2:**
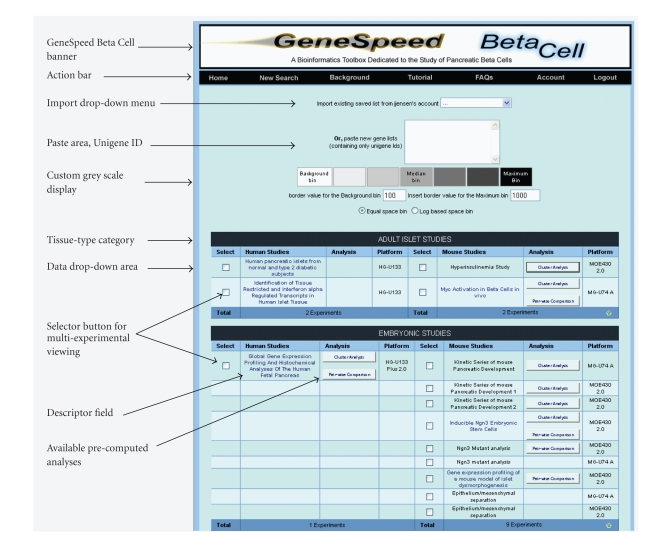
Web-page view of the GeneSpeed Beta Cell experiment selection window.

**Figure 3:**
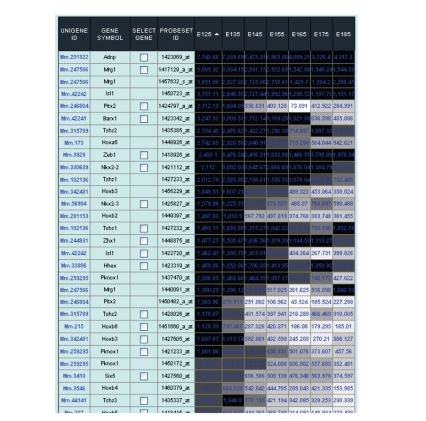
Example output page of homeodomain-class genes expressed in pancreatic development from E12.5 to E18.5. The list was ordered based on expression levels in E12.5 pancreas. As examples, Mrg1 and Adnp are expressed at high levels throughout pancreatic development. Several genes such as Pitx2 and multiple Hox-family members decrease abruptly after E14.5, and are only expressed significantly during early organ development.

“GeneSpeed Beta Cell” is a gene array data repository linked to the GeneSpeed environment. For a more detailed description of the domain-based gene categorization afforded by GeneSpeed, please refer to [3] and the online background and tutorials. GeneSpeed Beta Cell consists of a central “experiment selector page” (Figure 2), listing experiments by relevance to embryonic development, adult islet studies, adult whole pancreas studies, experiments using cell lines, and solid tumor data. Currently, the site is being expanded with gene chip data of developing nonpancreatic endoderm. For each group, experiments are separated into human and mouse studies. At current, Affymetrix-type data is supported, given that the body of relevant genomics data is the largest on this particular platform; but as the parent GeneSpeed database is not platform-specific, we have also made it capable of operating with the Illumina BeadChip type datasets. All current Affymetrix-type datasets in the repository were obtained as unnormalized raw cel files, and were normalized using MAS5.0 using identical settings and similarly scaled for cross-experimental comparisons (see methods). The available data can be viewed through multiexperimental viewing. Any saved gene list can be viewed for any of the available datasets. This is a fast and convenient way to display the normalized expression values of defined gene lists between independent experiments performed in different laboratories. The resulting display page is constructed to facilitate horizontal glancing of expression values, while maintaining the individuality of experiments. As there is no limit as to the number of genes shown or number of experiments selected, the resulting page view can be quite large. To assist the identification of the respective column (tissue type/experimental condition) and row (gene symbol), a hovering tool supplying this information was implemented. Also, for quick analysis of the gene ID, each cell is hyperlinked to the respective Unigene page for that gene. If a gene within a gene list does not contain a respective probeset, the cell content is displayed as N.A. The multiexperiment viewer facilitates table sorting based on each component in selected datasets. This provides, for example, quickly arranging genes in a larger gene list according to expression levels for any tissue/condition selected (e.g., Figure 3 shows a list of homeodomain-class genes sorted based on expression at the E12.5 gestational time point in pancreatic development).

### 2.2. Multidimensional analysis in “My Gene Workspace”

**Figure 4:**
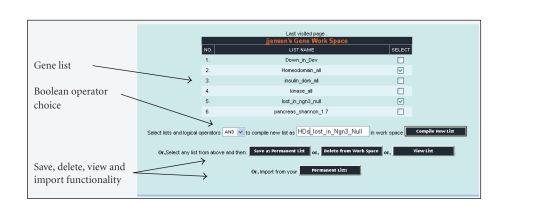
Web-page view of “My Gene Workspace” selection window.

To enhance online capabilities, we developed tools for multidimensional analysis. The multidimensional analysis tool operates within a “My Gene Workspace” environment (Figure 4), which is array-platform independent as it stores genes by Unigene identifier. “My Gene Workspace” allows for temporary storage of gene lists, naming such, and selecting individual lists to be combined using the Boolean operators AND or OR. Hereby, intersections (AND), or additive combinations (OR) can be performed on the selected gene lists, for further logical operations or visualized using the multiexperimental viewer. Several means of populating the workspace is possible. The user is provided with a “permanent list” account, in which work between sessions can be saved. Lists can here be grouped according to project name. Gene lists from the permanent account can be ported to the workspace or gene family choices from a concurrent GeneSpeed query that can be directly imported. In addition, gene list results from precalculated analyses based on the available datasets can be added. The final option provides a highly useful method to dynamically aggregate and compare results from individual experimental data that was not initially designed for a combined analysis. Such comparisons can be highly scientifically relevant, and examples are provided later.

As this latter method is based on precalculated analysis of available datasets, a certain level of a priori choice has been necessary to implement, as all permutations of possible data analysis could not be practically implemented. Consequently, depending on the underlying experimental conditions, the precomputed analysis is restricted to pairwise analysis (although multiple pairwise comparisons are often provided for a given dataset), or a self-organizing cluster analysis (for series-type data such as experimental time, drug concentration, or developmental time). Graphical presentation of each analysis is provided to help the user gauge gene numbers given the conditions chosen. For pairwise analysis, a volcano plot (plotting significance (*p*-value) versus fold-change) of the pairwise analysis result is shown. The default cutoff for gene selection is set at a false discovery rate of 0.1, but can be changed to the user's preference. Similarly, the fold-change range can be freely set, allowing the user to port, that is, >2-fold upregulated genes in a given condition into the workspace. The graphical presentation of cluster analyses contains a cluster number, and number of genes within the cluster. The user is free to select any number of clusters and port to the workspace. In this manner, various experimental conditions can be continuously ported to the workspace, and the experimental multi-intersectional analysis occurs there. There is no limit to the number of gene lists present in the workspace. We should note that for both the multidimensional viewing page, and for the multidimensional query form in the workspace, individual datasets are always kept separate (viewer), treated as such (query page), and are never pooled. Cross-experimental pooling is not tolerable due to varying conditions in different laboratories during data generation.

### 2.3. Current experimental content of GeneSpeed Beta Cell

As the available datasets and analyses grow on a daily basis, users should visit the site for a list of currently available datasets and analyses.

### 2.4. GeneSpeed Beta Cell use-case scenarios

Some biologically relevant use-case scenarios for the islet cell biologist are described in the following. Each of these is available also as online tutorials at GeneSpeed Beta Cell at http://genespeed.ccf.org/betaCell/tutorial.jsp. As for any bioinformatics-based method application, the results are provided as *candidate* gene lists, corresponding to genes/probesets fulfilling input criteria. The further validation of such lists using noninformatics-based techniques is a general requirement. In the following demonstrations, the end-result gene lists are often supported by previous published data from other sources, hereby providing the validation required for the particular demonstration scenarios.

**Example 1**

(Compiling lists of islet-expressed transcription factors (online tutorial 1)). We wish to address the issue of defining islet-expressed transcription factor (TF) encoding genes. To do this, we will utilize the predefined transcription factor categorization provided by the GeneSpeed database, assemble a nonredundant list of TF encoding genes, and find those reduced in *Ngn3* null pancreas. First, we select “new search,” and desired species “mouse” from the drop-down menu. Next, we select “search by transcription factor classification” within the GeneSpeed search options. As 5 major domain family groupings exist for the transcription factor type genes, we will need to iterate the following procedure for each, but will here limit the families to the “Basic,” “Beta-Scaffold,” and “HTH” superfamilies. These families contain, for example, the leucine zipper, bHLH, and homeodomain transcription factor families, but not the Zn-finger class. Selecting “Basic” as the first type, we ctrl-select all the subfamily members of the basic TF superfamily. Displaying the result provides 685 hits. These correspond to every instance where the Unigene database of the mouse contains a homology hit for any of the domain types associated the “basic” superfamily. However, as the database has no preset lower E-score cutoff, several false positives exist in this list (see discussion of how to set an E-score cutoff on the description pages at GeneSpeed for a full explanation). To eliminate low-scoring similarity hits, we set the E-score cutoff at E10-6, and redo the search. Now, a resulting list of 167 genes is detected. We save these to the user account under an arbitrary name (All_TFs). This process is repeated for the TF superfamilies mentioned above, where the individual results is added to the All_TF's list, consequently providing a list of >1600 individual Unigenes. These are next imported into the “My Gene Workspace.” To extract genes unique to pancreatic islets in the developing pancreas, we will take advantage of the available dataset for Ngn3-null embryonic pancreas, which is listed under the experiment listing page of GeneSpeed Beta Cell. A pair-wise analysis is provided comparing E15.5 Wt and E15.5 *Ngn3* Null pancreas. The *Ngn3*-deficient pancreas is excellent to define endocrine-specificity, as the organ is devoid of endocrine cells. Selecting genes upregulated >1.5 fold, *P* < .25, a second list is imported into the workspace as *Down_in_Ngn3*. This list contains 114 genes. Obtaining the intersection between the *TF_ALL* and *Down_in_Ngn3* lists provide a total of 8 transcription factor encoding genes lost in Ngn3 mutant E15.5 pancreas: *Ngn3, NeuroD, Isl1, Pax6, Arx, MafB, Nkx2.2, Insm1*, and *HIF1a*.

**Example 2**

(Multidimensional intersection analysis to define developmentally regulated expression of protein kinase-encoding genes (online tutorial 2)). We here will seek to discover kinase-encoding genes that are enriched in either early or late pancreatic development. A similar study has not been done before. To perform this task, we first need to compile a list of all protein kinase-type genes in the mouse transcriptome. Using a text-search for a gene known as a protein kinase (e.g., insr), we obtain two hits: *Insr* and *Insrr*. Both of these are receptor tyrosine kinases, and display the presence of the Tyr_pkinase domain (IPR001245) with an E-score at 1E10^−145^. We also note that the generic kinase domain (IPR000719) is detected in both at 1E10^−24^. By checking the “InterPro sub-search” box for the IPR001245 domain, and execute the search: “refine by subsearch,” we obtain a nonredundant list of Unigene clusters having similarity to the IPR001245 domain. This provides 480 hits, covering all kinase-domain forms (S/T as well as Y-kinase types). To curate against low-similarity hits, we manually set the E-score threshold at <1E-6. The resulting list contains bona-fide 432 kinase-containing genes, which we subsequently save as “*Kinase_all*” to our account. Many of these genes represent genes with no previous annotation as being of the kinase-domain containing type, and may not have been named yet. Next, we wish to identify which of these kinase-encoding genes display a downward trend during pancreatic development. To do this, we move to the “search GeneSpeed Beta Cell,” and expand the “Embryonic studies” dataset tab. Selecting the “kinetic series of mouse pancreatic development 1” precomputed cluster analysis, we are provided with the results of a Kohonen's self-organizing cluster analysis in a graphical format. Gene clusters with a downward trend during pancreatic development are selected (cluster 3,4,5,8,9,15,20) and combined using the selection tool provided. The results are saved as *Genes_Trend_Down* to the workspace. Within the workspace the intersection between the Genes_*Trend_Down* and the *Kinase_all* lists are obtained using the Boolean operator AND. The resulting list contains 138 kinase-type genes. The list can be saved, or gene expression of the particular genes can be displayed in some or all mouse array experiments in the GeneSpeed Beta Cell database. The latter may provide important clues as to tissue-specific expression of individual members. Finalizing this demonstration, we wish to address the identity of kinase-encoding genes that are upregulated over time in the developing pancreas. By repeating the above method for kinase-type genes displaying upregulation (Cluster 6,11,16,17,21,22, generating list *Gene_Trend_Up*), only 27 genes are identified. We can conclude that more kinase signaling diversity exists prior to rather than after the secondary transition in the mouse pancreas.

**Example 3**

(Defining human islet-specific expression using Shannon Entropy with exocrine elimination (online tutorial 3)). This example uses the available dataset on human tissues, as provided by the Novartis Genomics Institute (http://symatlas.gnf.org/SymAtlas/about.jsp). A tissue set consisting of 79 human tissues and 61 different mouse tissues, mostly adult solid organs, has been generated in duplicate using the Affymetrix GNF1 platform. To provide a measure of tissue expression selectivity, we adopted the method of Shannon entropy determination, as previously described by Schug et al. [4]. Shannon entropy provides quantitative measures of expression using a bit-rate scale. For each gene, the Shannon entropy (*H*_gene_) defines the degree of ordered expression; as a rule, the lower the *H*_gene_, the fewer tissues in the total set express the gene in question. To identify those tissues showing uniqueness in expression, the measure *Q*_tissue_ can be used. Again, as a rule, the lower the *Q*_tissue_ value, the more specific the gene is expressed in that particular tissue. A rank order of the lowest *Q*_tissue_ values thus provides a list of those genes that have the highest selectivity for the tissue in question. Shannon entropy computations were performed for all tissues in the above datasets. As the human, but not the mouse, datasets contain array data for islets, the present example is currently limited to human. From the GeneSpeed search query, we select species “homo sapiens,” and thereafter “search by expression.” We select the human GNF1A chip, and “calculated Shannon entropy” using the drop-down boxes. The following page contains selector boxes for each tissue in the set. For pancreatic islets, we select a *Q*_tissue_ value of < 1.7 × *H*_gene_. The user should have experiment with the *Q*_tissue_ setting; the lower values (approaching *H*_gene_) provide smaller numbers, but more tissue-specific genes. Relaxing the value towards a value of 2 × *H*_gene_ provides more exhaustive, albeit less selectively expressed genes. At *Q*_islet_ at 1.7 × *H*_gene_, we identify 96 probesets (as more than one probeset may exist for each gene, the actual number of genes identified is often slightly lower). Selecting to show all Shannon entropy values, we next copy the entire table into Excel, in order to rank-order the hits. Not surprisingly, the top of this list consists of Glucagon (*GCG*), Insulin (*INS*), *IAPP*, but we also notice that not far from the top, some nonendocrine-type genes, such as *PNLIPRP1* and *CPA2* are present. The reason for this is due to exocrine contamination. The majority of such genes are easily removed by eliminating all genes in which *Q*_panc_ < *Q*_islet_. Ranking the resulting genes provides a list of genes showing the highest selectivity for pancreatic islets compared to 78 other human tissues (the 30 top-ranked genes of this list is shown in Table 1). The results include the complement of endocrine terminal products (*Ins, Gcg, Sst, Ppy*), four Reg-type genes (*Reg1b, Reg3g, Reg3a, Regl*), several well-known endocrine transcripts (*Pcsk1* (PC1/3), *Iapp, Slc30a8*), secretogranins (*Scgb2a1, Scgn, Scg5, Scg2, Scg3*), and transcription factors known to function in the islet (*FoxA2, Nkx2.2, Isl1*).

**Example 4**

(Pituitary versus pancreatic islets: finding common neuroendocrine properties (online tutorial 4)). It is known that neuroendocrine cell types shares certain characteristics related to production and release of secreted products. The pituitary and islets are highly enriched in cells producing polypeptide hormones. Using Shannon entropy, we will here ask what are the genes that may be in common between pituitary and pancreatic islets and not expressed widely elsewhere. Similar to above, we select Shannon entropy query in GeneSpeed, and input a slightly relaxed *Q*_tissue_ value of 1.8 for both pituitary and pancreatic islets. Individually, *Q*_Islet_ < 1.8 × *H*_*g*_ and *Q*_pituitary_ < 1.8 × *H*_*g*_ identify 292 and 222 probesets, respectively. The intersection is 21 probesets, corresponding to 19 individual genes (Table 2, 3 probesets for *GNAS*, guanine nucleotide binding protein were identified). Three genes encode known granule-type proteins (*ChgrA*, secretogranin 2 (*SCG2*), secretogranin 5 (*SCG5*)). Two transcription factors are found: InsM1 and ZNF91. The proprotein convertase subtilisin/kexin type-1 inhibitor (*PCSKN1*) and the peptidylglycine alpha-amidating monooxygenase are also present. Other products include *CACNA1F* (Calcium channel, voltage-dependent, alpha 1F), *CNGA3* (Cyclic nucleotide gated channel alpha 3), the transmembrane protein *TMEM30* as well as several uncharacterized genes. Many of these genes represent expected hits, and show the value of combining parameters such as tissue uniqueness and overlapping gene expression to derive a meaningful candidate repertoire for further scrutiny.

## 3. DISCUSSION

Of the current available places for genomics data reposition, the NCBI GEO (gene expression omnibus, [5]) is presently the most exhaustive. The development of GEO proceeds to include data analysis of public array-type experiments, which also include those deposited on islets, or developing pancreas. The tools are currently limited to analyses performed within individual experiments, and data results cannot be ported between experiments. However, no other resource exists with a similar exhaustive compilation of DNA microarray-type datasets, and as such, GEO represents a growing and increasingly important pillar for array data compilation. In contrast to the more universal user-base that GEO seeks to cover, certain resources have also been made available and dedicated to the islet community. T1Dbase (http://www.t1dbase.org/) was specifically developed to catalogue information on the genetics of type-I diabetes, and contains extensive information on candidate gene regions [1]. It also contains a microarray repository and a recently developed *Gene Atlas* search function, aimed at providing a rapid visualization of gene expression in islets. The strength of the environment lies in the use of Gaggle [6], which is a Java-based communicator interface to several bioinformatics tools. However, to use this requires a significant knowledge of the Gaggle-implemented tools such as the TIGR tmev (http://www.tm4.org/mev.html) or R (http://www.r-project.org/), notwithstanding a rather complicated data upload scheme. Despite its strength, this may therefore represent a time-consuming and intellectual barrier to most biologists using the resource irregularly. Another comparable resource, the EpconDb (http://www.cbil.upenn.edu/epcondb42/) [2], originally generated by the Endocrine Pancreas Consortium and funded through the NIH Beta Cell Biology Consortium (http://www.betacell.org/), also provides microarray chip repository support. Recently, precalculated analysis results for select experiments are also provided. The structure of the EpConDb resource centers on the GUS (genome unified schema), which includes the DOTS database. DOTS shares significant similarities to the NCBI-devised Unigene EST database, but extend to include splice site data, as well as promoter definition.

The GeneSpeed Beta Cell site seeks to complement these resources on particularly two fronts: to provide more extensive orthogonal analysis between array experiments and to provide a functional gene list operator workspace, which neither the T1dbase nor Epcondb sites allow. To achieve the former, we focused on providing a larger degree of relevant precomputed analyses of array experiments providing these in an easy-to-query format. To achieve the latter, we developed a gene list workspace that would allow for platform-to-platform compatibility using the common Unigene denominator, which is the nexus of the GeneSpeed MySQL database. The current version of the database provides certain features not found elsewhere, some of which has been addressed through the demonstration cases. Yet, the database is a currently developing structure that in its present form is useful, but easily imagined improved. Therefore, we are currently focusing on key aspects for the further development of the GeneSpeed environment. These include the identification of additional relevant microarray experiments; filling out “missing links” by performing stop-gap-type microarray experiments for populating critical, but missing, areas of the pancreatic expression space; improving the search and query formats for user-friendliness; and finally developing an export/import interface for pathway analysis programs such as Ingenuity Pathway Analysis (IPA).

The usefulness of GeneSpeed Beta Cell database is dependent on the amount of available genomics data content. A linear increase in number of available datasets and accompanying precomputed analyses translates into an exponentially growing set of query combinations. There are obvious gaps in the available datasets, as multiple null mutations have been created for several key developmental regulators during pancreatic development, and several mutant models resulting in diabetes due to beta-cell dysfunction have also been reported, all of which would represent valuable data in the present environment. Therefore, we are asking the islet research community to share available datasets for multidimensional analysis. Also, we will continue to upload publicly available datasets from the GEO environment.

For a wet-biology laboratory like our own, the present incarnation of the database has provided means of moving forward in otherwise difficult-to-execute bioinformatics-based questions. We hope that the same appreciation may pioneer gene identification challenges in other laboratories hereby helping the diabetes research community.

## 4. METHODS

### 4.1. Genomics data incorporation and analysis

The “GeneSpeed Beta Cell” environment was developed using the J2EE platform on a Linux server. For Affymetrix-type genomics data, we compiled the CEL files (raw data) associated with different experiments from different sources and normalized them locally using MAS5.0 algorithm, using an identical scaling factor of 500, to ensure optimal comparability in a cross-experimental setting.

The microarray experiments currently available can be grouped, and hence analyzed, according to experimental design type. For time-series experiments (and drug-effect studies), an SOM neural network clustering algorithm was applied. The number of clusters selected is empirically based on individual results, selecting the minimal number adequately describing the data complexity. A graphical presentation is provided of the log-transformed expression averages of genes within the cluster. Also, the total number of gene number contained/cluster is provided. R and Bioconductor [7] were used to accomplish this task.

For multicomponent analysis, which also includes single pair-wise analysis, ANOVA testing was performed. For multiple-condition datasets, several pair-wise analyses are provided. These results are depicted through volcano plots. A false discovery rate (FDR) test correction on the ANOVA result at 10% significance is provided for each plot as the default *P*-value setting. On the volcano plots, the boxed areas outlining a 10% FDR corrected *P*-value and the −2 to +2 fold regions of change are shown.

### 4.2. Account functionality

There are two account types in GeneSpeed: “guest” and “registered user.” In order to use the workspace environment registration is required. A “registered user” can log back into their account to gain access to saved studies. Establishing a registered account is free and can be done on the GeneSpeed registration page (http://genespeed.ccf.org/loginReq.php). An automated password will be sent to the newly registered user. The confidentiality of all registration information is strictly maintained and we will only use such information to notify our users of any disruptions or modifications of the GeneSpeed service. At present, only registered users are allowed to use the GeneSpeed Beta Cell database.

The GeneSpeed account allows registered users to save gene lists into a private account that is permanent and may only be viewed by the owner. The “My Gene Workspace,” on the other hand, maintains gene lists temporarily during the current login session; upon logging out the content of the “workspace” will be deleted.

### 4.3. Functional implementation of “My Gene Workspace”

The “My Gene Workspace” logic was developed using J2EE and sql-type queries. Facilitating cross-platform comparisons, the workspace utilizes Unigene cluster Ids (UID). Consequently, the probeset identification through the experimental analyses is translated into corresponding UID upon transfer to the workspace. As a result, if more than one probeset is detected for a given gene in the analysis, these probesets collapse into the UID of that gene. Secondly, upon selection of the content of a gene list in the workspace, followed by showing the content in the expression space, all probesets corresponding to the selected Unigene will be displayed. To reduce ambiguities, we update the system continuously upon the availability of updated mapping files from NetAffx Analysis Center server. Given that the NCBI Unigene dataset is constantly evolving, updated mapping to the most recent UID is done every 6 months.

## Figures and Tables

**Table 1:** Results, use-case scenario 3. Genes defined as most specific for human islets as based on Shannon Entropy calculations.

Gene	Symbols	Entropy *H*_*g*_	*Q*_Islets_	*Q*_Pancreas_
Hs.516494	GCG	1.32	1.65	4.22
Hs.89832	INS	1.2	1.74	3.04
Hs.46835	IAPP	1.95	2.33	5.63
Hs.4158	REG1B	2.82	3.91	4.48
Hs.447084	REG3G	3.61	4.46	7.89
Hs.567312	REG3A	3.45	4.65	5.41
Hs.584797	REGL	3.61	4.91	5.59
Hs.12409	SST	4	5.15	9.27
Hs.558368	PPY	4.28	5.36	11.82
Hs.78977	PCSK1	4.31	5.72	11.8
Hs.97644	SCGB2A1	4.17	5.83	12.14
Hs.204238	LCN2	3.95	6.24	9.34
Hs.612083	NA	4.23	6.51	7.43
Hs.73923	PNLIPRP1	4.69	6.57	7.05
Hs.489786	CFTR	4.8	6.85	7.29
Hs.116428	SCGN	5.03	6.89	9.34
Hs.76452	CRP	5.18	7.21	8.66
Hs.156540	SCG5	4.92	7.62	11.4
Hs.53985	GP2	5.23	7.71	7.72
Hs.516726	SCG2	4.96	8.3	12.7
Hs.232618	SCG3	5.12	8.49	13.3
Hs.534458	CGI-38	5.02	8.51	13.37
Hs.125898	GNAS	5.64	8.6	12.81
Hs.8364	PDK4	5.72	8.62	10.84
Hs.592742	ELL2	5.54	8.63	12.26
Hs.2256	MMP7	5.7	8.7	11.14
Hs.123072	RAB3B	5.13	8.78	13.24
Hs.155651	FOXA2	5.45	8.95	9.62
Hs.516922	NKX2-2	5.45	9.17	12.68
Hs.558519	ERO1LB	5.63	9.18	11.22
Hs.491232	SLC39A14	5.55	9.18	10.11
Hs.260720	DNAJC12	5.69	9.25	11.76
Hs.82071	CITED2	5.58	9.36	13.37
Hs.503733	LOC653275	5.88	9.38	11.61
Hs.203699	GOLPH3L	5.59	9.41	11.95
Hs.479602	APBB2	5.76	9.45	13.23
Hs.109590	GENX-3414	5.89	9.79	13.27
Hs.89655	PTPRN	6.01	9.84	12.71
Hs.532270	SLC30A8	5.97	9.87	12.33
Hs.505	ISL1	6	9.9	11.7

**Table 2:** Results, use-case scenario 4. Genes specific for both pancreatic islets and pituitary.

	Unigene ID	Symbol	Gene name
1	Hs.6790	DNAJB9	DnaJ (Hsp40) homolog, subfamily B, member 9
2	Hs.156540	SCG5	Secretogranin V (7B2 protein)
3	Hs.516726	SCG2	Secretogranin II (chromogranin C)
4	Hs.389378	MON2	MON2 homolog (S. cerevisiae)
5	Hs.150793	CHGA	Chromogranin A (parathyroid secretory protein 1)
6	Hs.152944	LOH11CR2A	Loss of heterozygosity, 11, chromosomal region 2, gene A
7	Hs.631626	ZNF91	Zinc finger protein 91
8	Hs.89584	INSM1	Insulinoma-associated 1
9	Hs.234785	CNGA3	Cyclic nucleotide gated channel alpha 3
10	Hs.632799	CACNA1F	Calcium channel, voltage-dependent, alpha 1F subunit
11	Hs.369430	PAM	Peptidylglycine alpha-amidating monooxygenase
12	Hs.146180	TMEM30B	Transmembrane protein 30B
13	Hs.125898	GNAS	GNAS complex locus
14	Hs.459183	ALPK3	Alpha-kinase 3
15	Hs.87295	FAM18B	Family with sequence similarity 18, member B
16	Hs.522640	PCSK1N	Proprotein convertase subtilisin/kexin type-1 inhibitor
17	Hs.496542	RNF128	Ring finger protein 128
18	Hs.444459	C9orf135	Chromosome 9 open reading frame 135
19	Hs.503733	LOC653275	Similar to cryptic/cripto

## References

[1] Hulbert EM, Smink LJ, Adlem EC (2007). T1DBase: integration and presentation of complex data for type 1 diabetes research. *Nucleic Acids Research*.

[2] Mazzarelli JM, Brestelli J, Gorski RK (2007). EPConDB: a web resource for gene expression related to pancreatic development, beta-cell function and diabetes. *Nucleic Acids Research*.

[3] Kutchma A, Quayum N, Jensen J (2007). GeneSpeed: protein domain organization of the transcriptome. *Nucleic Acids Research*.

[4] Schug J, Schuller WP, Kappen C, Salbaum JM, Bucan M, Stoeckert CJ (2005). Promoter features related to tissue specificity as measured by Shannon entropy. *Genome Biology*.

[5] Barrett T, Troup DB, Wilhite SE (2007). NCBI GEO: mining tens of millions of expression profiles—database and tools update. *Nucleic Acids Research*.

[6] Shannon PT, Reiss DJ, Bonneau R, Baliga NS (2006). The Gaggle: an open-source software system for integrating bioinformatics software and data sources. *BMC Bioinformatics*.

[7] Reimers M, Carey VJ (2006). Bioconductor: an open source framework for bioinformatics and computational biology. *Methods in Enzymology*.

